# Geographical Correlation between Refined Sugar Consumption and Oral Cancer Incidence: A Global Ecological Study: Correlation between Sugar Consumption and Oral Cancer

**DOI:** 10.31661/gmj.v14i.3789

**Published:** 2025-03-16

**Authors:** Mohammad Jafariheydarlou, Negar Sarrafan

**Affiliations:** ^1^ Department of Oral and Maxillofacial Disease, School of Dentisity, Urmia University of Medical Science, Urmia, Iran; ^2^ Department of Oral and Maxillofacial Medicine, School of Denticity, Urmia University of Medical Science, Urmia, Iran

**Keywords:** Refined Sugar Consumption, Oral Cancer Incidence, Global Ecological Study

## Abstract

**Background:**

Refined sugar consumption is a known risk factor for metabolic disorders and has been linked to some cancers. However, its potential role in oral cancer remains poorly understood. Oral cancer significantly contributes to global cancer-related mortality and is driven by dietary, behavioral, and socioeconomic factors. This ecological study investigates the global correlation between refined sugar intake and oral cancer incidence, accounting for major confounders.

**Materials and Methods:**

We analyzed publicly available data from the World Health Organization (WHO) and World Bank databases. The primary variables included oral cancer incidence per 100,000 population and sugar consumption. Statistical analyses included descriptive statistics, Pearson correlation coefficients, and multiple linear regression models to assess associations and adjust for confounding factors.

**Results:**

The study analyzed data from multiple countries, revealing substantial regional variation in oral cancer incidence and refined sugar consumption. Correlation analysis showed a weak negative association between refined sugar consumption and oral cancer incidence (r=-0.05, P0.05). Also, Multiple linear regression confirmed refined sugar consumption was not associated with oral cancer (β=-0.0028, P=0.606).

**Conclusion:**

This study did not identify a significant association between sugar consumption and oral cancer incidence at the population level. However, the absence of a detectable relationship in this ecological analysis does not preclude more complex mechanisms or indirect associations. For example, sugar’s role in promoting metabolic disorders, which might interact with other risk factors like inflammation or oxidative stress, warrants further study. Public health efforts should prioritize tobacco and alcohol reduction, given their robust and well-documented links to oral cancer, while future research explores the broader health impacts of dietary sugars.

## Introduction

Oral cancer represents a significant global health burden, accounting for substantial morbidity and mortality worldwide. It encompasses malignancies originating in the lips, tongue, gums, floor of the mouth, and other oral cavity regions [1][2]. According to the World Health Organization (WHO), oral cancer ranks among the top 15 most prevalent cancers globally, with an annual incidence exceeding 370,000 cases [3]. The relationship between dietary habits and cancer incidence has drawn increasing attention in epidemiological research over recent decades. Among various lifestyle factors, the role of refined sugar consumption in cancer development has sparked particular interest due to its widespread inclusion in modern diets and its links to metabolic diseases.

Refined sugar is a concentrated form of sucrose, often derived from sugarcane or sugar beets, and is commonly added to processed foods and beverages.

The link between high sugar intake and metabolic disorders, such as obesity and diabetes, is well-established. However, evidence for its direct role in oral cancer development is limited and warrants further investigation.

Recent research has underscored the potential association between high-sugar diets and multiple cancer types, attributing this link to their contribution to chronic inflammation and cellular damage, both of which facilitate carcinogenesis [4].

Moreover, emerging ecological studies have explored regional variations in dietary patterns, particularly sugar consumption, to identify potential correlations with cancer incidence [5]. While such studies have successfully mapped correlations between high-sugar consumption and increased incidences of colorectal, breast, and other cancers, research specifically targeting oral cancer remains limited [6][7]. This gap highlights the necessity for studies examining geographic and dietary correlations specific to oral cancer, which can provide valuable insights into potential preventive measures [8].

Ecological studies examining geographical dietary patterns have provided insights into public health risks associated with certain diets, such as diets high in refined sugars. The regions with high per capita sugar consumption, particularly in North America and parts of Europe, have reported notable oral cancer incidence rates [9]. Although these correlations may be influenced by additional environmental or behavioral factors, studying these dietary trends geographically could reveal patterns relevant to oral cancer risk [10].

This study aims to explore the geographical correlation between refined sugar consumption and oral cancer incidence across various regions using an ecological research approach. By analyzing available data on per capita sugar intake and oral cancer rates, we aim to identify patterns that may suggest a relationship between these variables. The findings of this study have the potential to inform public health policies targeting dietary interventions and contribute to the broader understanding of modifiable risk factors in oral cancer prevention.

## Materials and Methods

**Table:** Table1. Descriptive Statistics of Variables

**Variables**	**Mean**	**SD**	**Minimum**	**25th Percentile**	**Median**	**75th Percentile**	**Maximum**
Oral Cancer per 100 K	2.77	2.29	0.4	1.4	1.9	3.6	21.2
Per capita refined Sugar Consumption (g/day)	69.30	34.02	7.4	44.275	69.05	94.575	148.2
Current tobacco use prevalence (%)	19.29	9.06	3.2	11.525	19.55	25.2	45.3
Alcohol (liters)	4.33	3.48	0	1.33	3.35	7.21	12.64
GDP per capita (USD)	13718.36	19536.64	216.82	1877.1	5308.16	16707.62	116905.37
Prevalence of obesity among adults (%)	20.71	11	1.7	10.37	21.1	28.92	59.9
Rural population (%)	40.90	22.43	0	21.9	40.5	57.8	86.7
Population (M)	44.68	155	0.17	3.34	10.47	33.27	1411.1

**SD:** standard deviation; **K:**1000 people; **M:** Million; **USD:** Us Dollar ($)

**Figure-1:**
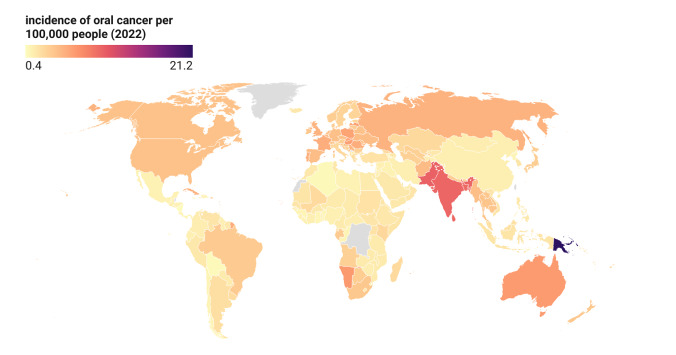
Figure 1. World map illustrating the distribution of oral cancer incidence rates, with a focus on the including these countries: Gambia, Nicaragua, Congo, Brunei Darussalam, Bolivia, Algeria, Jamaica, Democratic People's Republic of Korea, Benin, Panama, Ghana, Guinea, Armenia, Eswatini, Bahamas, Chile, Comoros, Democratic Republic of the Congo, Dominican Republic, Honduras, Iraq, Lebanon, Libya, Mexico, Costa Rica, Ecuador, Guinea-Bissau, Liberia, Maldives, Nigeria, Sierra Leone, Syrian Arab Republic, Timor-Leste, China, Cote d'Ivoire, Guatemala, Guyana, Iran, Jordan, North Macedonia, Rwanda, Barbados, Central African Republic, Colombia, El Salvador, Haiti, Mongolia, Tajikistan, Togo ,Zimbabwe, Cameroon, Mozambique, Paraguay, United Arab Emirates, Zambia, Bahrain, Burundi, Chad, Egypt, Israel, Kuwait, Malta, Niger, United Republic of Tanzania, Burkina Faso, Malawi, Peru, Philippines, Saudi Arabia, Trinidad and Tobago, Tunisia, Yemen, Albania, Azerbaijan, Cyprus, Djibouti, Ethiopia, Iceland, Kyrgyzstan, Lesotho, Morocco, Suriname, Eritrea, Oman, Senegal, South Sudan, Venezuela, Vietnam, Indonesia, Mauritania, Republic of Korea, Somalia, Sudan, Turkey, Uganda, Malaysia, Qatar, Argentina, Equatorial Guinea, Singapore, Greece, Solomon Islands, Uruguay, Vanuatu, Madagascar, Mali, Georgia, Kazakhstan, Italy, Kenya, Fiji, Bhutan, Estonia, Finland, Mauritius, Samoa, Bosnia and Herzegovina, Sweden, Turkmenistan, Angola, Bulgaria, Uzbekistan, Austria, Japan, Nepal, Botswana, Norway, Brazil, Denmark, Gabon, Lithuania, Lao People's Democratic Republic, New Zealand, South Africa, Cambodia, Slovenia, Switzerland, Ireland, Thailand, Belarus, Canada, Croatia, Luxembourg, Saint Lucia, United States of America, Czechia, Germany, Netherlands, Montenegro, Myanmar, Republic of Moldova, Spain, Ukraine, Afghanistan, Belgium, Serbia, Russian Federation, United Kingdom of Great Britain and Northern Ireland, Cuba, Portugal, France, Romania, Cabo Verde, Latvia, Poland, Slovakia, Hungary, Australia, Namibia, Bangladesh, Sri Lanka, India, Pakistan, Papua New Guinea. (sorted from the lowest to the highest incidence)

This ecological study examines the relationship between refined sugar consumption and oral cancer incidence across multiple geographical locations, utilizing data from publicly available and reputable sources.

### Data Sources

1. World Health Organization (WHO) Global Health Observatory (GHO): Data on oral cancer incidence rates, tobacco use prevalence, alcohol consumption, obesity prevalence, and screening programs were obtained from the WHO Global Health Observatory database. This data set provides health-related metrics across countries, which allows for cross-regional comparisons of lifestyle factors associated with oral cancer incidence. Available here: https://www.who.int/data/gho

2. World Bank Database: Data on economic indicators, including Gross Domestic Product (GDP) per capita and rural population percentages, were obtained from the World Bank database. This source provided standardized economic and demographic indicators across countries, facilitating an analysis of socioeconomic factors potentially associated with health outcomes. Available here: databank.worldbank.org

### Data Extraction and Cleaning

Data were extracted from the WHO and World Bank databases in CSV and Excel formats. The data were collected for the most recent year available, typically between 2018 and 2023, ensuring consistency across datasets. All included:

• Oral Cancer Incidence per 100,000 Population: The primary dependent variable, representing the incidence rate of lips and oral cavity cancer across countries.

• Refined Sugar Consumption (grams per capita per day): As an independent variable, this factor was explored for its potential association with oral cancer.

• Tobacco Use Prevalence (%): Estimate of current tobacco use prevalence (%)

• Alcohol Consumption (liters per capita): Annual alcohol consumption per capita, standardized for adults aged 15 and older in liters of pure alcohol.

• Obesity Prevalence (%): Prevalence of obesity among adults, body mass index (BMI) more or equal to 30 (crude estimate) (%), represented as a percentage as an indirect index of sugar consumption.

• Screening Programs (Binary): Indicator of the presence or absence of oral health screening for early detection of oral diseases.

• Gross domestic product (GDP) per Capita (US Dollar): It is the economic output of a nation per person and is used as a socioeconomic indicator.

• Population: The number of individuals residing in the country during the year 2022.

• Rural Population (%): Rural population percentage was included as a variable to explore its potential influence on access to healthcare and early detection rates, which may indirectly impact oral cancer incidence.

The cleaning process addressed inconsistencies and missing values:

• Range Values: Variables provided in ranges (e.g., tobacco use prevalence and obesity prevalence) were standardized by extracting the mean of the given range to represent a single value per country.

• Missing Data: Columns with high levels of missing values (e.g., tobacco use in some regions) were examined, and imputation or exclusion was performed as appropriate for maintaining data integrity.

### Data Quality and Potential Biases

The quality of the data used in this study is an important consideration when interpreting the findings. Although the analysis utilizes globally recognized data sources, including the WHO Global Health Observatory (GHO) and World Bank databases, several limitations and potential biases must be addressed.

Oral cancer incidence rates may be influenced by inconsistencies in reporting standards and diagnostic practices across countries. In regions with limited healthcare infrastructure or underdeveloped cancer registries, oral cancer cases may be underreported or misclassified. Conversely, high-income countries with advanced healthcare systems likely have more accurate and comprehensive reporting, which can create geographical biases in the data. Similarly, data on refined sugar consumption is based on national-level estimates, which may not fully capture individual-level consumption patterns or variations within countries.

Imputation and Missing Values: To address missing data, imputation methods or exclusions were applied, which may introduce bias if the missingness is not random. Countries with missing data on key variables, such as tobacco use, might systematically differ from those with complete datasets, potentially affecting the generalizability of the results.

Temporal Mismatch in Data: While the study primarily used data from 2022, some variables reflect data from earlier years due to availability constraints. This temporal mismatch could distort findings if risk factor prevalence or health outcomes have shifted over time, such as changes in sugar consumption patterns or tobacco use trends.

Aggregated Nature of Variables: National averages for variables like sugar consumption and obesity prevalence fail to account for within-country heterogeneity. Rural and urban populations, for example, may exhibit vastly different dietary patterns, risk factor exposures, and access to healthcare, which this study cannot capture.

Implications for Interpretation: These data limitations underscore the importance of cautious interpretation. The study provides valuable insights into global patterns but cannot establish individual-level causality or capture nuanced variations. Future research should prioritize longitudinal and individual-level data to validate and expand upon these findings. Additionally, efforts to standardize data collection and reporting across countries would significantly enhance the reliability and comparability of global studies.

### Statistical Analysis

Descriptive Statistics: Descriptive statistics were calculated for all variables, including measures of central tendency (mean, median) and dispersion (standard deviation, minimum, and maximum values). These summaries provided an initial understanding of the variability and distribution of key health and lifestyle factors across countries.

Correlation Analysis: Pearson correlation coefficients were computed to evaluate associations between oral cancer incidence and other variables, including sugar consumption, tobacco use, alcohol consumption, obesity prevalence, and GDP per capita. This correlation matrix enabled the identification of potential factors influencing oral cancer incidence.

Multiple Linear Regression: A multiple linear regression model was developed with oral cancer incidence as the dependent variable and refined sugar consumption as the primary independent variable. Additional covariates, such as GDP per capita, rural population percentage, tobacco use prevalence, alcohol consumption, and obesity prevalence, were included to control for confounding effects. This model assessed the strength and significance of each factor in predicting oral cancer incidence, with coefficients interpreted to understand the direction and magnitude of associations.

### Software

Data analysis and visualization were performed using Python (version 3.11) with packages including Pandas for data manipulation, Seaborn and Matplotlib for visualization, and Stats models for regression analysis.

## Results

**Table:** Table2. Pearson Correlation Coefficients and Multiple Linear Regression Analysis Results

**Variable**	**r**	**Coefficient**	**SE**	**t-Statistic**	**P-Value**	**95% CI (Lower)**	**95% CI (Upper)**
Intercept	-	-0.19	0.82	-0.24	0.81	-1.83	1.44
Per capita refined Sugar Consumption (g/day)	-0.05	-0.002	0.005	-0.52	0.60	-0.01	0.008
Current tobacco use prevalence (%)	0.39	0.10	0.02	5.11	<0.01*	0.06	0.14
Alcohol consumption (litres of pure alcohol)	0.27	0.14	0.05	2.58	0.01*	0.03	0.25
Prevalence of obesity among adults (%)	-0.006	-0.02	0.01	-1.16	0.25	-0.05	0.01
GDP per capita (USD)	0.18	0	0	3.05	0.003*	0	0
Rural population (% of total population)	-0.075	0.01	0.01	1.4	0.16	-0.006	0.03

**r:** Pearson Correlation Coefficient; **SE:** Standard error; ^*^Significant (P-Value<0.05)

**Figure-2:**
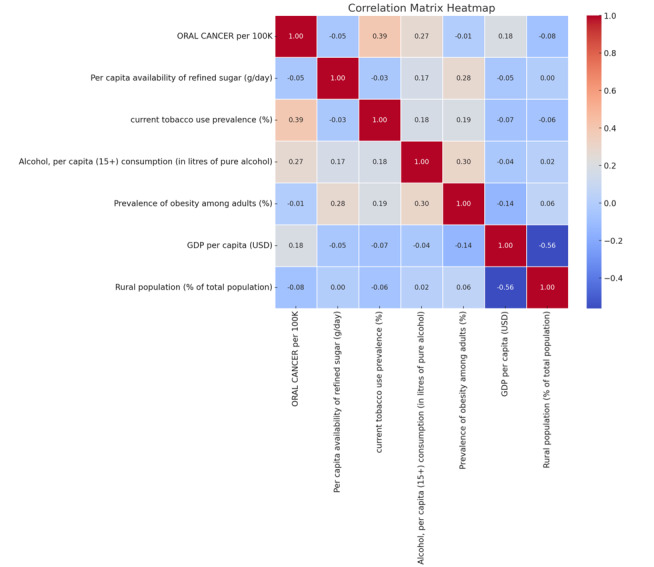
Figure 2. The Correlation Matrix Heatmap highlights the relationships among variablesF

**Figure-3:**
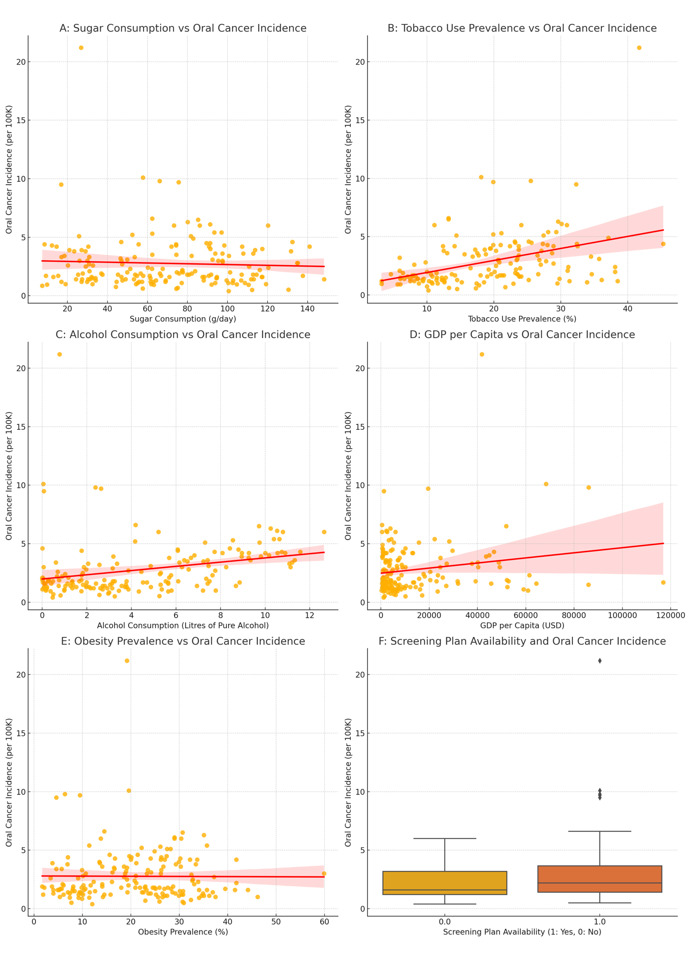
Figure 3. The relationship between oral cancer rates and A: Sugar consumption per capita, B: Tobacco Use Prevalence, C: Alcohol consumption, D: GDP per capita, E: Prevalence of obesity. Regression Line: The trend line shows a very slight negative slope, which aligns with the regression results indicating no significant relationship between sugar consumption and oral cancer incidence. Data Points: The scattered distribution of points supports that there isn’t a strong or consistent pattern between these two variables in this dataset. F: Screening plan availability, a binary variable, is visualized using boxplots. Each boxplot displays the distribution of oral cancer incidence rates for countries grouped by the presence or absence of a screening plan.

### Descriptive Statistics

Descriptive statistics for all primary variables are presented in Table-1. The average incidence of oral cancer across the countries studied was 2.77 ± 2.29 cases per 100,000 individuals, from 0.4 to 21.4. Figure-1. shows the global distribution of oral cancer incidence.

Refined sugar consumption averages 69.3 ± 34 grams per person per day, reflecting significant variability in dietary habits. Sugar consumption ranges from as low as 7.4 grams to 148 grams per person per day. GDP per capita also showed a broad range, reflecting economic diversity among the countries included. Also, 73.42% of countries had established screening programs for oral disorders.

### Correlation Analysis

Pearson correlation coefficients were calculated to evaluate associations between oral cancer incidence and refined sugar consumption, along with other potential covariates. Table-2 shows the strength and direction of correlations with oral cancer incidence and Figure-2 highlights the relationships between variables.

Sugar consumption demonstrated a weak negative correlation with oral cancer incidence (r=-0.05, P>0.05), suggesting no strong population-level association. However, these findings are exploratory and should be interpreted with caution given the ecological nature of the study.

Tobacco use prevalence showed a moderate positive correlation with oral cancer incidence (r=0.39, P<0.01), indicating a possible link between smoking and oral cancer risk. Alcohol consumption also demonstrated a positive correlation (r=0.27, P<0.05), suggesting that higher alcohol intake may be associated with increased oral cancer incidence. GDP per capita exhibited a weak positive correlation (r=0.18, P<0.05), while obesity prevalence and rural population percentage were not significantly correlated with oral cancer incidence.

### Multiple Linear Regression Analysis

A multiple linear regression supported correlation analysis. Figure-2 highlights the relationships among variables in a matrix heatmap. The regression model explained 24.7% of the variance in oral cancer incidence (R²=0.247, P<0.001). Detailed regression results are presented in Table-2, while Figure-3 illustrates the relationships among key variables and oral cancer incidence. The multiple linear regression analysis revealed no significant association between refined sugar consumption and oral cancer incidence in the adjusted model (β=-0.0028, P=0.606). While this suggests that sugar consumption may not directly influence oral cancer risk, the possibility of indirect effects mediated through other factors cannot be ruled out. As expected, tobacco use and alcohol consumption demonstrated stronger positive associations with oral cancer, reinforcing their established roles as primary risk factors. GDP per capita was positively associated with oral cancer incidence. Also, rural population percentage and obesity prevalence were not significant predictors, implying a limited impact on oral cancer risk after controlling for other factors.

## Discussion

This study assessed the potential relationship between refined sugar consumption and oral cancer incidence across multiple regions. Despite hypothesized links between high sugar intake and cancer risks [5], our findings did not reveal a significant association between refined sugar consumption and oral cancer incidence. This finding prompts a closer examination of relevant research on sugar intake and cancer, as well as possible mechanisms through which refined sugar could influence, or fail to influence, oral cancer risk [11].

While several studies have examined the relationship between refined sugar intake and cancer risk, research specific to oral cancer is sparse. Most existing literature highlights indirect mechanisms, such as sugar's role in promoting obesity and metabolic syndrome, which are associated with elevated risks for some cancers. However, these pathways may not directly influence oral cancer, given its distinct etiology involving carcinogens like tobacco and alcohol [4][5]. Moreover, Zainal et al, [12] identified that dietary sugar facilitates the growth of cancer cells in in-vitro studies. In contrast, there are a few hypothesized mechanisms by which refined sugar could theoretically influence cancer risk, such as increased oxidative stress and inflammation resulting from high blood glucose levels. However, evidence connecting these mechanisms specifically to oral cancer is limited [6].

While the large cohort study suggested a protective link between sugar intake and oral cancer risk in men [13], the in-vitro study highlighted sugar's role in enhancing tumor growth and chemoresistance during treatment [14].

These contrasting findings underscore the complexity of sugar's effects on cancer and the importance of context. On the other hand, our study found no significant relationship between sugar consumption and oral cancer, suggesting that the metabolic pathways commonly associated with refined sugar intake may not have a direct influence on oral carcinogenesis [5]. This finding is in line with other studies that failed to observe a direct connection between sugar intake and oral cancer but found stronger associations with cancers related to the gastrointestinal and endocrine systems, where metabolic disruption plays a more central role [15].

Another consideration is the nature of refined sugar as a dietary factor that often coexists with other lifestyle risk factors. For instance, sugar intake is sometimes correlated with obesity and poor diet quality, which can contribute indirectly to cancer risk [11].

However, as our study found no significant association between refined sugar consumption and oral cancer, nor between obesity prevalence and oral cancer incidence, it suggests that sugar intake alone, independent of broader dietary or lifestyle patterns, does not play a significant role in oral carcinogenesis [5]. On the other hand, in oral tissues, direct carcinogens like tobacco are more prominently linked to DNA damage and cellular changes than dietary sugars, which may explain the stronger association with tobacco observed in this study and others [16]. Our findings highlight the influence of tobacco and alcohol use on oral cancer incidence, alongside an unexpected inverse relationship with obesity prevalence.

The positive correlation between tobacco use and oral cancer incidence (r=0.39) aligns with extensive research identifying tobacco as a primary risk factor for oral cancer, with its carcinogenic compounds directly damaging oral epithelial cells and promoting malignancy [16].

Also, Alamgir et al. reported significantly higher oral cancer risks among long-term tobacco users, reinforcing the need for public health initiatives to reduce tobacco consumption [17]. On the other hand, in our study, alcohol showed a weaker but positive correlation with oral cancer incidence (r=0.27), paralleling studies that suggest alcohol may contribute to carcinogenesis by damaging mucosal cells and acting synergistically with tobacco [18]. However, its effect remains secondary to tobacco, with mixed results across populations [19].

Moreover, the weak but significant association between GDP per capita and oral cancer incidence reflects broader patterns in healthcare accessibility and diagnostic rates in higher-income countries. While GDP itself is not a direct risk factor, it likely represents confounding variables such as screening program prevalence and access to medical care [20][21]. However, our evaluation, like the study by Purkayastha et al, [22] found no evidence of an impact of early detection plans on oral cancer prevalence. This finding underscores the multifaceted nature of oral cancer risk, where socioeconomic status may modulate risk via lifestyle factors rather than serve as a primary risk determinant.

### Implications and Limitations

As an ecological study, the findings are subject to the ecological fallacy, where associations observed at the population level may not apply to individuals. For instance, a country with high sugar consumption and low oral cancer incidence does not imply that individuals consuming more sugar are at lower risk. Also, we did not measure the confounding effect of human papillomavirus (HPV) as a risk factor for oropharynx cancer because it is not available in the WHO database, however, due to vacation the prevalence of it significantly decreased [19]. Overall, these limitations prevent causal inferences and highlights the need for individual-level studies to validate these findings.

## Conclusion

This study found no significant population-level association between refined sugar consumption and oral cancer incidence, suggesting that its direct role in oral carcinogenesis may be limited. However, further research, including longitudinal and individual-level studies, is necessary to explore potential mechanisms and indirect effects. These findings reaffirm the importance of addressing tobacco and alcohol use as primary risk factors for oral cancer. These findings support existing research, reaffirming that modifiable lifestyle factors, particularly tobacco and alcohol use, are the strongest predictors of oral cancer risk. Our findings emphasize prioritizing interventions for tobacco and alcohol reduction.

## Conflict of Interest

None.
